# The COVID‐19 pandemic and associated declines in cancer incidence by race/ethnicity and census‐tract level SES, rurality, and persistent poverty status

**DOI:** 10.1002/cam4.70220

**Published:** 2024-09-13

**Authors:** Benmei Liu, Mandi Yu, Jeffrey Byrne, Katheen A. Cronin, Eric J. Feuer

**Affiliations:** ^1^ Division of Cancer Control and Population Sciences National Cancer Institute Bethesda Maryland USA; ^2^ Information Management Services, Inc. Calverton Maryland USA

**Keywords:** cancer incidence, census tract‐level groups, COVID‐19, health disparity, pandemic

## Abstract

**Background:**

The COVID‐19 pandemic had a significant impact on cancer screening and treatment, particularly in 2020. However, no single study has comprehensively analyzed its effects on cancer incidence and disparities among groups such as race/ethnicity, socioeconomic status (SES), persistent poverty (PP), and rurality.

**Methods:**

Utilizing the recent data from the United States National Cancer Institute's Surveillance, Epidemiology, and End Results Program, we calculated delay‐ and age‐adjusted incidence rates for 13 cancer sites in 2020 and 2015–2019. Percent changes (PCs) of rates in 2020 compared to 2015–2019 were measured and compared across race/ethnic, census tract‐level SES, PP, and rurality groups.

**Results:**

Overall, incidence rates decreased from 2015–2019 to 2020, with varying PCs by cancer sites and population groups. Notably, NH Blacks showed significantly larger PCs than NH Whites in female lung, prostate, and colon cancers (e.g., prostate cancer: NH Blacks −7.3, 95% CI: [−9.0, −5.5]; NH Whites: −3.1, 95% CI: [−3.9, −2.2]). Significantly larger PCs were observed for the lowest versus highest SES groups (prostate cancer), PP versus non‐PP groups (prostate and female breast cancer), and all urban versus rural areas (prostate, female breast, female and male lung, colon, cervix, melanoma, liver, bladder, and kidney cancer).

**Conclusions:**

The COVID‐19 pandemic coincided with reduction in incidence rates in the U.S. in 2020 and was associated with worsening disparities among groups, including race/ethnicity, SES, rurality, and PP groups, across most cancer sites. Further investigation is needed to understand the specific effects of COVID‐19 on different population groups of interest.

## 1 INTRODUCTION

The COVID‐19 pandemic, caused by severe acute respiratory syndrome coronavirus 2 (SARS‐COV‐2), emerged to the world starting from late 2019.^1^ Since the first cases of COVID‐19 were diagnosed in the United States in late January 2020, this new virus has caused substantial increases in mortality among patients that peaked in late‐Spring 2020 and Winter 2020/2021.^2^

The pandemic has caused disruptions in cancer screening, diagnosis, and treatment.^3^, ^4^, ^5^, ^6^, ^7^, ^8^ The number of people getting screened for some cancer types in the U.S. dropped dramatically, especially in the first few months of the pandemic. For example, the May 2020 EPIC study, based on electronic health record (EHR) data, showed that preventive cancer screenings in the U.S. abruptly dropped by 86% for colon cancer and 94% for breast and cervical cancers following the declaration of the COVID‐19 national emergency.^8^ By July 2020, the number of cancer screenings had begun to rise but had not yet reached previously expected levels.^9^ Using data from the population‐based Behavioral Risk Factor Surveillance System,^10^ Fedewa et al. estimated that past‐year breast cancer screening and cervical cancer screening decreased by 6% (from 61.6% to 57.8%) and 11% (from 58.3% to 51.9%) from 2018 to 2020, respectively, while the past‐year colorectal cancer screening rate remained steady between 2018 and 2020 because past‐year stool testing increased by 7%, offsetting a 16% decrease in colonoscopy.^3^ The number of people getting screened for cancer could have dropped for a few reasons, including temporal facility closure, staffing shortages, social distancing measures, etc. A few studies have reported the impact of the pandemic on diagnosis of new cancers, resulting in large decreases in new cancer diagnosis.^7^, ^11^, ^12^ Using a forecasting modeling approach, Burus et al. estimated that approximately 134 thousand new cancer diagnosis could be missed in the U.S. during the first 10 months of the pandemic.^13^

The pandemic has further exacerbated disparities in health care.^14^ Serval studies observed racial and ethnic disparities on cancer care, indicating that racial and ethnic minority groups were more likely to be infected with COVID‐19 and were more likely to have pandemic‐related delays in cancer care than White patients.^15^, ^16^, ^17^ A few studies examined associations between the pandemic and disparities in cancer incidence.^13^, ^18^ However, there is not a single study that has comprehensively evaluate how cancer incidence changed in 2020 across many cancer sites and many subgroups facing disparities.

Using recent population‐based cancer incidence data, this paper examines the COVID‐19 pandemic and associated declines in cancer incidence and related changes in disparities across race ethnicity groups, socio‐economic status (SES), rurality, and persistent poverty (PP) groups categorized at the census‐tract level.

## 2 METHODS

### 2.1 Data sources

Population‐based cancer incidence data were obtained from the U.S. National Cancer Institute's Surveillance, Epidemiology, and End Results (SEER) Program November 2022 submission (SEER 22 excluding Alaska and Illinois)^19^ for the diagnosis years of 2015–2019 combined and 2020 for 13 selected cancer sites (some are further stratified by sex). The SEER registries included Connecticut, Atlanta, Greater Georgia, Rural Georgia, San Francisco‐Oakland, San Jose‐Monterey, Greater California, Hawaii, Idaho, Iowa, Kentucky, Los Angeles, Louisiana, Massachusetts, New Mexico, New Jersey, New York, Seattle‐Puget Sound, Texas, and Utah, covering about 44% of the U.S. population. The population covered by SEER is comparable to the general U.S. population with regard to measures of poverty (15.3% vs. 15.1% of below poverty level) and education (14.2% vs. 13.0% of less than high school diploma among persons aged 25 and more). These registries were selected for inclusion in the SEER program based on their ability to operate and maintain a high‐quality population‐based cancer reporting system and for their epidemiologically significant population subgroups.^20^ The data includes information on individuals' sex, racial/ethnic group, and aggregate level SES, PP status, and rurality for the census tract of residence. Census tracts are small geographic areas with approximately 4000 population, that are designed to be relatively homogenous with respect to the characteristics of the individuals residing there. They are much more informative than using counties as the geographic unit because counties vary greatly in size, and within county differences become homogenized when utilizing aggregate level characteristics.

The cancer sites of interest contained five cancer sites where screening is recommended, including female breast, prostate, lung and bronchus (female and male), colon and rectum, and cervix uteri, and two others where early detection through incidental or other types of findings frequently occurs, i.e., thyroid (female and male) and melanoma, even though screening is not currently recommended. Six other cancer sites were also included where screening or early detection is not available, namely pancreas, liver and intrahepatic bile duct, urinary bladder, kidney, and renal pelvis, ovarian, and corpus and uterus—NOS. The data from the diagnosis years 2015 to 2019 were combined to reduce statistical variability.

The racial/ethnic groups include Non‐Hispanic (NH) White, NH Black, NH American Indian and Alaska Native (AIAN) residing in Purchased/Referred Care Delivery Areas (PRCDA), NH Asian or Pacific Islander (API) and Hispanic. The census‐tract level SES groups of interest included the lowest and the highest quintile groups based on the SES Yost index quintiles weighted by census tract population (i.e., approximately equal size populations in each quintile).^21^ The rurality groups of interest included two categories: 100% urban (all urban) and rural (100% rural or mostly rural, i.e., >0% but <50% urban), which utilized three categories of the Urban Rural Indicator Codes (URIC).^22^ The URIC is an urban–rural classification variable constructed from the 2010 U.S. Census's percent of the population living in non‐urban areas with four categories (all urban, mostly urban, mostly rural, all rural tracts). Rural population encompasses all population not included within an urban area.^23^ For this project, the two rural groups (mostly rural and all rural) were combined due to small population sizes. Tracts with PP are those that have had at least 20% of their residents living below the federal poverty line since 1980.^24^ About 11.4% of the SEER population live in census tracts with PP. All the information needed were accessed from the NCI's SEER*Stat Research Plus Limited‐Field Specialized Database.^25^

### 2.2 Statistical Analysis

For each of the 13 cancer sites (with lung and thyroid further stratified by sex) selected, delay‐ and age‐adjusted cancer incidence rates per 100,000 population (denoted as R) for each of the two diagnosis periods (2015–2019 and 2020) and the rate ratios of 2020 over 2015–2019, along with the 95% confidence intervals (CI), were computed for the five racial/ethnic groups, the two SES groups (lowest and highest), the two rurality groups (All urban and rural), the two persistent poverty groups (PP and non‐PP), and the four groups formed by PP status crossing by rurality using the SEER*Stat software.^25^ The 95% confidence intervals were estimated using Tiwari's method.^26^ United States 2000 standard populations by 5‐year age groups were used for computing age‐adjusted rates.^27^ The initially reported case count for a given diagnosis year tends to be smaller than the updated counts later reported for that year, which produces bias in estimating cancer incidence trends. Statistical methods using historical patterns of updated case counts have been developed in order to estimate “delay‐adjusted” counts and rates.^28^ Delay factors by racial‐ethnic groups, age at diagnosis, and registry were used.

We calculated the percent change (PC) of the adjusted cancer incidence rates between diagnosis year 2020 and 2015–2019 for each cancer and group of interest. For example, for female breast cancer, the PC of incidence rate (2020 vs. 2015–2019) for NH White individuals is defined as:

$$\begin{array}{c} {\mathrm{PC}}_{2020/1519}^{\mathrm{NHW}}=100\%\times \left(R_{2020}^{\mathrm{NHW}}-R_{1519}^{\mathrm{NHW}}\right)/R_{1519}^{\mathrm{NHW}}\\ =100\%\times \left({\mathrm{RR}}^{\mathrm{NHW}}-1\right), \end{array}$$

where $R_{2020}^{\mathrm{NHW}}$ and $R_{1519}^{\mathrm{NHW}}$ are the age‐adjusted rate for NH Whites in 2020 and 2015–2019 respectively, and ${{\mathrm{RR}}^{\mathrm{NHW}}=R}_{2020}^{\mathrm{NHW}}/R_{1519}^{\mathrm{NHW}}$ is the rate ratio of the age‐adjusted incidence rate of 2020 over 2015–2019 for NH Whites. The corresponding 95% CI of ${\mathrm{PC}}_{2020/1519}^{\mathrm{NHW}}$ was calculated as follows:

$$\begin{array}{c} {\text{Lower}\_\text{bound}\_\mathrm{PC}}_{2020/1519}^{\mathrm{NHW}}\\ =100\%\times \left(\text{Lower}\_\text{bound}\_{\mathrm{RR}}^{\mathrm{NHW}}-1\right), \end{array}$$

$$\begin{array}{c} {\text{Upper}\_\text{bound}\_\mathrm{PC}}_{2020/1519}^{\mathrm{NHW}}\\ =100\%\times \left(\text{Uper}\_\text{bound}\_{\mathrm{RR}}^{\mathrm{NHW}}-1\right), \end{array}$$

where the lower and upper bound of ${\mathrm{RR}}^{\mathrm{NHW}}$ were calculated using SEER*Stat software.

Similarly, the PC for the NH Black individuals is:

$$\begin{array}{c} {\mathrm{PC}}_{2020/1519}^{\mathrm{NHB}}=100\%\times \left(R_{2020}^{\mathrm{NHB}}-R_{1519}^{\mathrm{NHB}}\right)/R_{1519}^{\mathrm{NHB}}\\ =100\%\times \left({\mathrm{RR}}^{\mathrm{NHB}}-1\right), \end{array}$$

where $R_{2020}^{\mathrm{NHB}}$ and $R_{1519}^{\mathrm{NHB}}$ are the age‐adjusted rate for NH Blacks in 2020 and 2015–2019 respectively, and ${{\mathrm{RR}}^{\mathrm{NHB}}=R}_{2020}^{\mathrm{NHB}}/R_{1519}^{\mathrm{NHB}}$ is the rate ratio of the age‐adjusted incidence rate from 2020 over 2015–2019 for NH Blacks. The 95% CI of ${\mathrm{PC}}_{2020/1519}^{\mathrm{NHB}}$ was calculated as:

$$\begin{array}{c} {\;\text{Lower}\_\text{bound}\_\mathrm{PC}}_{2020/1519}^{\mathrm{NHB}}\\ =100\%\times \left(\text{Lower}\_\text{bound}\_{\mathrm{RR}}^{\mathrm{NHB}}-1\right), \end{array}$$

$$\begin{array}{c} {\;\text{Upper}\_\text{bound}\_\mathrm{PC}}_{2020/1519}^{\mathrm{NHB}}\\ =100\%\times \left(\text{Uper}\_\text{bound}\_{\mathrm{RR}}^{\mathrm{NHB}}-1\right). \end{array}$$

If the 95% CI of a PC is not crossing zero, then the PC is significantly different from zero (p‐value<0.05). These PCs reflect associations between the pandemic and changes in cancer incidence by comparing the rates between 2020 and the combined years 2015–2019.

To assess the statistical significance of these PCs between two contrasting population groups, we examined the overlap of their respective 95% confidence intervals.^29^ If the upper bound of the 95% CI of the smaller PC is less than the lower bound of the 95% CI of the larger PC in comparison, i.e., the two 95% CIs are not crossing, then the two PCs are significantly different from each other (p‐value <0.05). For example, for female breast cancer, to compare the PCs between NH White and NH Black, we looked at the overlapping of the confidence intervals associated with ${\mathrm{PC}}_{2020/1519}^{\mathrm{NHW}}$ and ${\mathrm{PC}}_{2020/1519}^{\mathrm{NHB}}$. Non‐overlap represents a conservative test of statistical significance.

## 3 RESULTS

**FIGURE 1:**
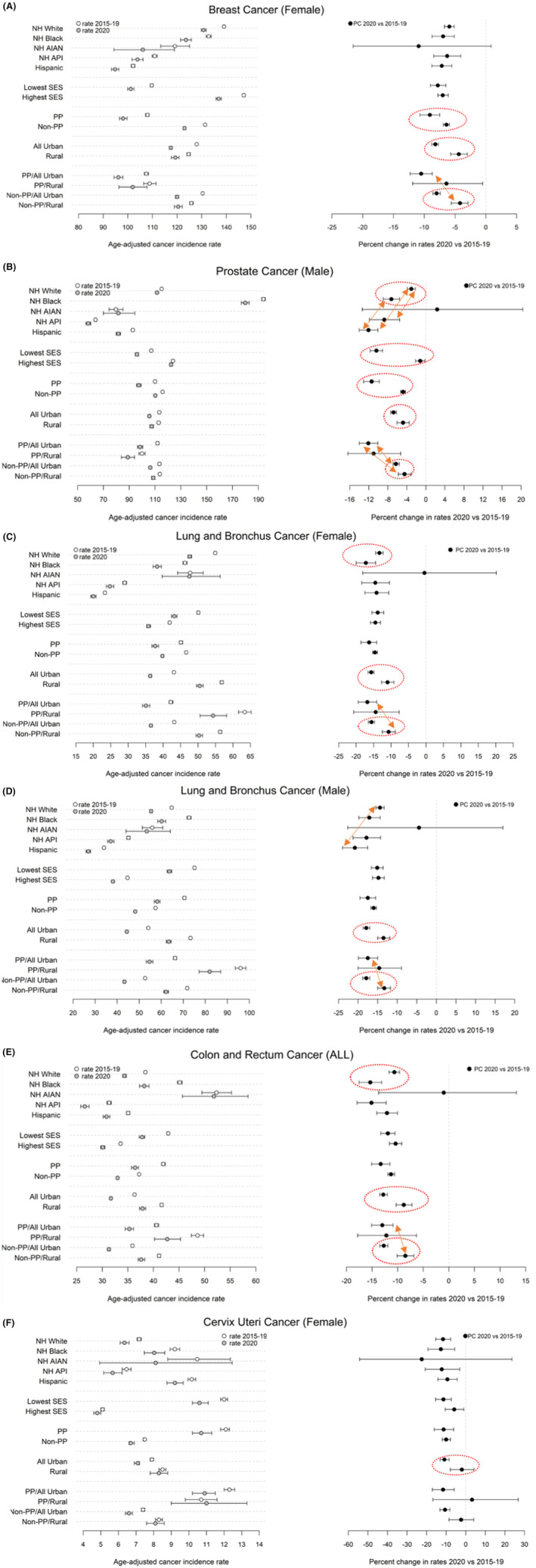
(A) Delay & age‐adjusted incidence rate (/100,000) and percent difference between 2020 and 2015–2019 for female breast cancer (cancer sites where screening is recommended). Groups with significant different PC were highlighted with dashed circles or arrows. (B) Delay & age‐adjusted incidence rate (/100,000) and percent difference between 2020 and 2015–2019 for prostate cancer (cancer sites where screening is recommended). Groups with significant different PC were highlighted with dashed circles or arrows. (C) Delay & age‐adjusted incidence rate (/100,000) and percent difference between 2020 and 2015–2019 for female lung and bronchus cancer (cancer sites where screening is recommended). Groups with significant different PC were highlighted with dashed circles or arrows. (D) Delay & age‐adjusted incidence rate (/100,000) and percent difference between 2020 and 2015–2019 for male lung and bronchus cancer (cancer sites where screening is recommended). Groups with significant different PC were highlighted with dashed circles or arrows. (E) Delay & age‐adjusted incidence rate (/100,000) and percent difference between 2020 and 2015–2019 for colon and rectum cancer (cancer sites where screening is recommended). Groups with significant different PC were highlighted with dashed circles or arrows. (F) Delay & age‐adjusted incidence rate (/100,000) and percent difference between 2020 and 2015–2019 for cervix uteri cancer (cancer sites where screening is recommended). Groups with significant different PC were highlighted with dashed circles or arrows.

**FIGURE 2:**
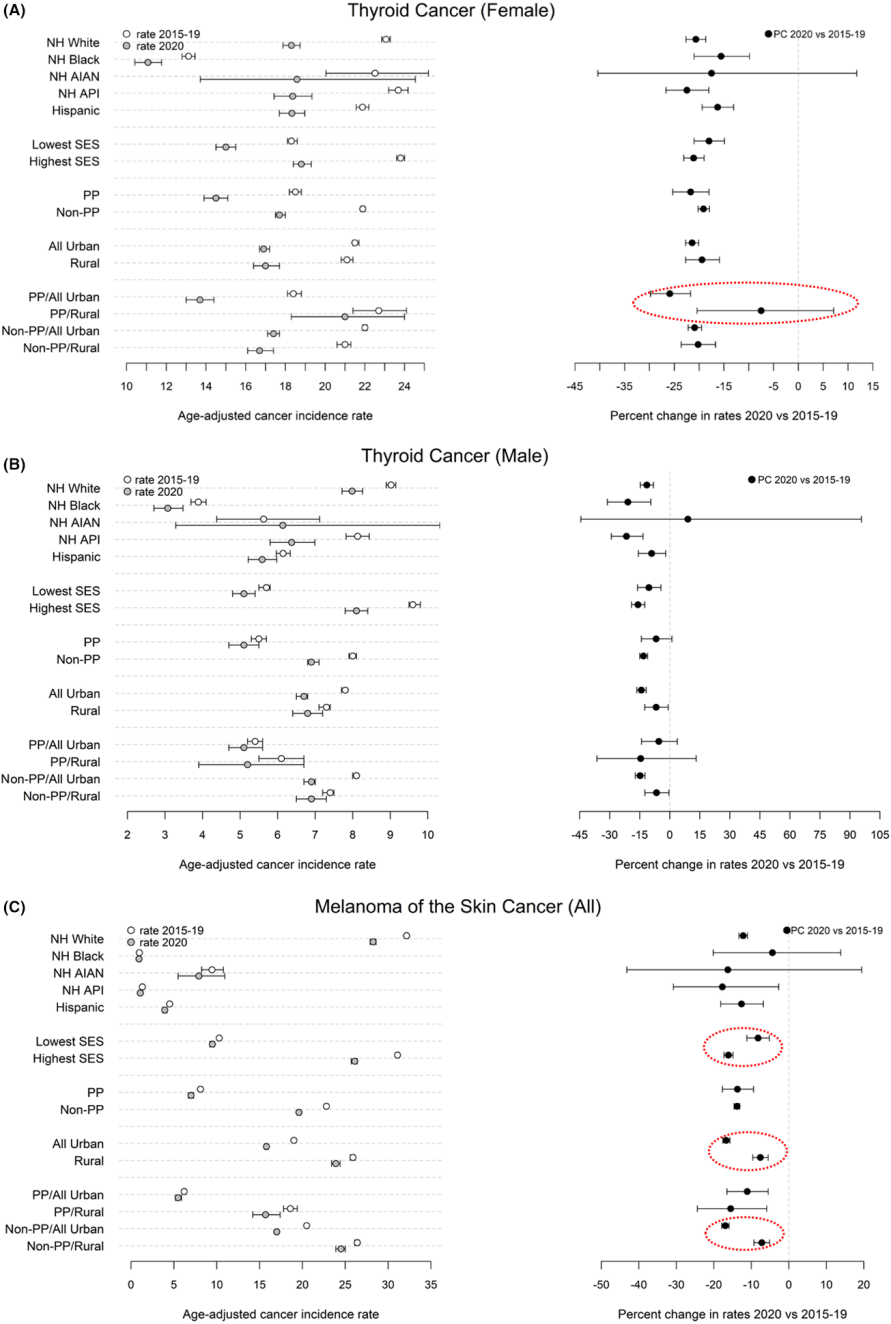
(A) Delay & age‐adjusted incidence rate (/100,000) and percent difference between 2020 and 2015–2019 for female thyroid cancer (cancer sites where early detection frequently occurs). Groups with significant different PC were highlighted with dashed circles or arrows. (B) Delay & age‐adjusted incidence rate (/100,000) and percent difference between 2020 and 2015–2019 for male thyroid cancer (cancer sites where early detection frequently occurs). (C) Delay & age‐adjusted incidence rate (/100,000) and percent difference between 2020 and 2015–2019 for melanoma of the skin cancer (cancer sites where early detection frequently occurs). Groups with significant different PC were highlighted with dashed circles or arrows.

**FIGURE 3:**
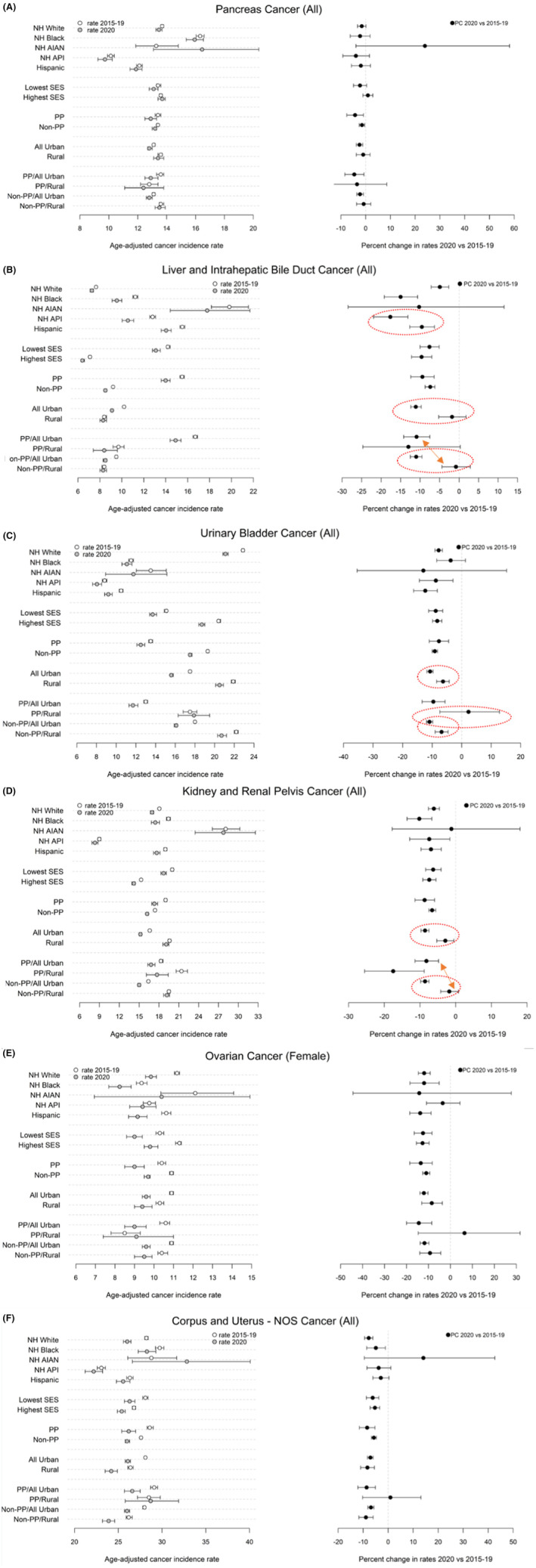
(A) Delay & age‐adjusted incidence rate (/100,000) and percent difference between 2020 and 2015–2019 for pancreas cancer (cancer sites where screening or early detection is not available). (B) Delay & age‐adjusted incidence rate (/100,000) and percent difference between 2020 and 2015–2019 for liver and intrahepatic bile duct cancer (cancer sites where screening or early detection is not available). Groups with significant different PC were highlighted with dashed circles or arrows. (C) Delay & age‐adjusted incidence rate (/100,000) and percent difference between 2020 and 2015–2019 for urinary bladder cancer (cancer sites where screening or early detection is not available). Groups with significant different PC were highlighted with dashed circles or arrows. (D) Delay & age‐adjusted incidence rate (/100,000) and percent difference between 2020 and 2015–2019 for kidney and renal pelvis cancer (cancer sites where screening or early detection is not available). Groups with significant different PC were highlighted with dashed circles or arrows. (E) Delay & age‐adjusted incidence rate (/100,000) and percent difference between 2020 and 2015–2019 for ovary cancer (cancer sites where screening or early detection is not available). (F) Delay & age‐adjusted incidence rate (/100,000) and percent difference between 2020 and 2015–2019 for corpus and uterus—NOS cancer (cancer sites where screening or early detection is not available).

The delay‐ and age‐adjusted cancer incidence rates from the two data points (2015–2019, 2020) and the percent change between the two data points are presented in figures by cancer sites which are classified into three groups (screening is recommended; early detection frequently occurs; screening or early detection is not available). The left panel of each figure presents the incidence rates, the right panel of each figure presents the PCs (Figures 1A–F, 2A–C, 3A–F). The detailed results are presented in two supplement tables. Table S1 presents the results by race/ethnicity and the two SES groups for all the 13 selected sites. Table S2 presents the results by PP status, rurality, and PP status crossing by rurality for all the 13 cancer sites.

### 3.1 Changes in Cancer Incidence from 2015–2019 to 2020

The delay‐ and age‐adjusted cancer incidence rates significantly decreased in 2020 compared to 2015–2019 for all the race/ethnicity groups (non‐overlap confidence intervals) other than NH AIAN for each of the following cancers: female breast, prostate, female and male lung and bronchus, colon and rectum (all), cervix, female and male thyroid, and liver (all) cancer (Table S1 and the left panel of Figures 1A–1F, 2A,B, 3B). The “all” in the parentheses followed a cancer site name refers to female and male combined. For example, among NH Whites, the incidence rates of female breast cancer in 2015–2019 and 2020 were 139.0 (95% CI: [138.6, 139.5]) and 130.8 (95% CI: [129.8, 131.8]) respectively. Decreases for the remaining cancer sites except pancreas (all) cancer only occurred in three or less racial ethnic groups: bladder (all) cancer for NH Whites, NH APIs, and Hispanics (Figure 3C); kidney (all) and ovarian cancer for NH Whites, NH Blacks, and Hispanics (Figure 3D); melanoma of the skin (all) cancer for NH Whites and Hispanics (Figure 2C); corpus and uterus—NOS only for NH Whites (Figure 3F). Pancreas (all) cancer had no significant change between 2015–2019 and 2020 for any of the racial ethnic groups (Figure 3A).

The delay‐ and age‐adjusted cancer incidence rates significantly decreased in 2020 across all the selected cancer sites for both the lowest and highest SES groups, except for prostate and cervix cancer. The decrease in prostate cancer incidence rate in the highest SES group (from 123.9 in 2015–2019 to 122.4 in 2020) and that in cervix cancer (from 5.1 in 2015–2019 to 4.8) were not statistically significant (Table S1).

The decreases in cancer incidence rates from 2015–2019 to 2020 were significant across all the cancer sites for people living in all‐urban census tracts, in non‐PP tracts, and the combination of all‐urban and non‐PP tracts (Table S2 and left panel of Figures 1, 2, 3). For example, the female breast cancer incidence rate decreased from 128.0 (95% CI: [127.7, 128.3]) in 2015–2019 to 117.5 (95% CI: [116.8, 118.1]) in 2020 for those living in the all‐urban tracts. Similar decreases were observed for the non‐PP tracts. For rural tracts, the decrease in incidence rates from 2015–2019 to 2020 was significant for all the selected cancer sites except male thyroid, cervix uteri, pancreas (all), liver (all) and kidney (all) cancer. For rural tracts with PP, the decreases were significant only for five cancer sites including prostate, female and male lung and bronchus, colon and rectum (all), melanoma of the skin (all) cancer. For rural tracts that are non‐PP, the decreases in incidence rates were significant for all the cancer sites except male thyroid, cervix, pancreas (all), and liver (all) cancer. The decreases were significant across all the cancer sites for tracts that were 100% urban and with PP except male thyroid and pancreas (all) cancer.

### 3.2 Comparing Percent Changes in Incidence Rate Across Population Groups

Among racial/ethnic groups, we found that PCs of incidence rates in 2020 compared to 2015–2019 were significantly larger for some population groups than for others across different cancer type. Specifically, the magnitude of PC (2020 vs. 2015–2019) of incidence rates for NH Blacks was significantly larger than that of NH Whites for prostate cancer (−7.3, 95% CI: [−9.0, −5.5] vs. −3.1, 95% CI: [−3.9, −2.2]), female lung and bronchus cancer (−17.2, 95% CI: [−20.0 to −14.40] vs. −13.3, 95% CI: [−14.38 to −12.3]), and colon and rectum (all) cancer (−15.4, 95% CI: [−17.5 to −13.1] vs. −10.6, 95% CI: [−11.7 to −9.6]) respectively (right panel of Figure 1B,C,E; Table S1). The PC for Hispanics was also significantly larger than that of NH Whites for prostate cancer (−12.1, 95% CI: [−14.0 to −10.1] vs. −3.1, 95% CI: [−3.9, −2.2]) and for male lung and bronchus cancer (−20.8, 95% CI: [−24.0 to −17.6] vs. −14.4, 95% CI: [−15.5 to −13.3]) (right panel of Figure 1B,D and Table S1).

No significant PC differences between the lowest and the highest SES groups were observed for all of the cancer sites except prostate and melanoma of the skin (all) cancer. For prostate cancer, a significantly larger PC was observed in the lowest SES group compared to the highest SES group (−10.4, 95% CI: [−11.7, −9.1] vs. −1.2, 95% CI: [−2.2, −0.2]) (right panel of Figure 1B). However, for melanoma of the skin (all) cancer, the highest SES group had significantly larger PC compared to the lowest SES group (−16.1, 95% CI: [−17.3, −14.9] vs. −8.2, 95% CI: [−11.2, −5.2]) (right panel of Figure 2C and Table S1).

Significantly larger PCs were observed in the PP tracts than that of the non‐PP tracts for female breast (−9.1, 95% CI: [−10.7, −7.5] vs. −6.4, 95% CI: [−6.9, −5.9]) and prostate cancer (−11.4, 95% CI: [−13.1, −9.8] vs. −4.8, 95% CI: [−5.3, −4.3]) (right panel of Figure 1A,B, and Table S2). Ten cancer sites had significantly larger PCs in the all‐urban tracts than those of the rural tracts. These cancer sites included: female breast, prostate, female and male lung and bronchus, colon and rectum (all), cervix, melanoma of skin (all), liver (all), bladder (all), and kidney (all) cancer (right panel of Figures 1A–F, 2C, 3B–D, Table S2). For example, for female breast cancer, the PC in the all‐urban tracts and the rural tracts was −8.2 (95% CI: [−8.8, −7.7]) and −4.4 (95% CI: [−5.7, −3.0]) respectively.

When comparing across groups formed by the interaction of PP status and rurality, within non‐PP tracts, significant larger PCs were observed in all‐urban tracts compared to rural tracts for female breast, prostate, female and male lung and bronchus, colon and rectum (all), liver (all), bladder (all), and kidney (all) cancer (right panel of Figures 1A–E, 3B–D). Within PP tracts, only female thyroid cancer had significantly larger PC in tracts that are all urban compared to those that are rural (−25.9, 95% CI [−29.8, −21.7] vs. −7.5, 95% CI: [−20.4, 7.1]) (right panel of Figure 2B). Several cancer sites also had significantly larger PC in tracts that are PP and all‐urban than those that are non‐PP and rural (female breast, prostate, female and male lung and bronchus, colon and rectum (all), liver (all) and kidney (all) cancer) (right panel of Figures 1A–E, 3B,D). For urinary bladder cancer, larger PC were also observed in the tracts that are non‐PP and all‐urban than that of tracts that are PP and rural (−10.9, 95% CI: [−11.9, −9.8] vs. 2.3, 95% CI: [−7.4, 12.8]) (right panel of Figure 3C).

## 4 SUMMARY AND DISCUSSION

Using the recent population‐based cancer registry data based on April 2023 data release, we examined the COVID‐19 pandemic and associated temporal decreases in cancer incidence across various population groups based on race/ethnicity, and census tract‐level SES, rurality, and PP status. To date, the census tract‐level characteristics for the 2021 data from the latest data release (April 2024) are not available yet. We compared the diagnosis year 2020 to the combined data from 2015–2019 for 13 selected cancer sites, including 5 sites where screening is recommended, 2 sites where early detection frequently occurs and 6 other sites. More significant percent changes of rates among population groups were found in the cancer sites where screening is recommended. There are several reasons why the pandemic might contribute to declines in cancer incidence rates, including delaying screening, delaying diagnostic confirmation of a positive screening test, and delaying follow‐up of clinical symptoms that could be associated with specific cancers.

While other publications have reported decreases in cancer diagnosis associated with COVID‐19 (e.g., see^12^, ^13^, ^18^), this analysis goes further by considering the association in the context of pre‐COVID‐19 levels for each population subgroup being considered. The PC in incidence offers a measure that is comparable across subgroups and provides insight into how the pandemic was associated with relative measures of disparities. Some notable differences in the percentage of changes between counterpart subgroups were observed. Those differences in the magnitude of PCs may indicate distinct behaviors between these groups impacted by COVID‐19. Significantly larger PCs for NH Blacks or Hispanics compared to NH Whites were observed for prostate, female and male lung and bronchus, and colon and rectum (all) cancers, these might indicate that NH Blacks and Hispanics were more affected by COVID‐19 in terms of cancer screening and/or early diagnosis. Significantly larger PCs in the PP tracts compared to non‐PP tracts were only observed for female breast and prostate cancer. However, majority of the cancer sites had significant larger PCs in all‐urban tracts compared to rural tracts. These observations may indicate that all‐urban areas were affected more by COVID‐19 than the rural areas. Prostate and melanoma of the skin (all) are the only two cancer sites that significant PC differences were observed in the lowest versus highest SES groups, but with a different direction. Larger PC was observed in the lowest SES for prostate cancer and in the highest SES group for melanoma of the skin (all) cancer. This inconsistency may be explained by the disproportionate distributions of race/ethnicity for the two cancer sites, that is, more NH Blacks with prostate cancer and more NH Whites with melanoma of the skin cancer. Further investigation is necessary to explain the varying associations between the COVID‐19 and cancer incidence across different racial/ethnic groups, SES levels, rurality, and PP status for different cancer sites. Taken together, these results suggest that the effects of the pandemic may exacerbate downstream health disparities in cancer mortality among NH blacks and Hispanics relative to NH whites, those living in areas with PP, and those living in urban areas. Evaluating PC will help to explain mortality difference seen in years to come.

In the context of screening, a pandemic related larger percentage decrease in incidence for one population subgroup relative to another might actually increase health disparities with respect to population mortality for the first group. For example, sharpless,^30^ using cancer modeling and some reasonable assumptions about COVID associated delays in screening for breast and colorectal cancer in 2020, showed that over the next decade almost 10,000 excess deaths from breast and colorectal cancer deaths; about a 1% increase from what we would expect if these delays had not occurred.

To our knowledge, this study is among the first to comprehensively analyze changes in cancer incidence and disparities before and during the COVID‐19 pandemic across a broad range of cancer sites and subgroup populations. The use of census‐tract level SES, rurality, and PP status accounted for the higher homogeneity within the population groups compared to those at county‐level. The statistical approaches used in this research are similar to those used in Semprini et al,^18^ which compared head and neck cancer incidence in U.S. before (2019) and during the pandemic (2020) by age, sex, race and ethnicity. However, we used combined 5‐year data (2015–2019) prior to the pandemic to increase statistical power and examined many more cancer sites and population subgroups. Our approaches differ from the recent Burus et al. study,^13^ which compared observed cancer incidence with predicted ones derived from forecasting models for March to December 2020.

The study has some limitations. We did not incorporate cancer screening data or other sources to provide further context for the observed patterns. In addition, the 2020 data only includes a single year, resulting in insufficient statistical power for small groups like NH AIAN individuals or smaller cancer sites.

The strong temporal associations of the early stages of COVID‐19 pandemic in 2020 with changes in health disparities in terms of cancer incidence may be multifaceted, including delayed screenings and diagnosis, disproportionate effects on marginalized populations, variations in healthcare utilizations, and psychological and emotional impact. The COVID‐19 pandemic resulted in disruptions in healthcare systems, including temporary closures, reduced capacity, and resource prioritization for COVID‐19 patients, leading to delays in cancer screenings, diagnostic tests, and treatment for many individuals. These delays in early detection can contribute to later‐stages cancer diagnoses, potentially affecting treatment outcomes and survival rates.^8^ Marginalized or disadvantaged communities, such as racial/ethnic minorities, low‐income individuals, and those with limited access to healthcare, have experienced a disproportionate burden of COVID‐19 cases and related consequences. These communities often face pre‐existing disparities in cancer incidence and outcomes and have been further exacerbated by the pandemic.^31^ Factors such as limited access to healthcare facilities, socioeconomic challenges, and systemic barriers have contributed to disparities in cancer care and outcomes. Additionally, the pandemic has resulted in shifts in healthcare utilization patterns, as individuals opt to delay or avoid medical visits due to concerns about virus transmission or healthcare system strain.^32^ This decrease in healthcare utilization may impact cancer detection rates and lead to missed opportunities for early intervention. Furthermore, the COVID‐19 pandemic has caused significant psychological distress and emotional strain on individuals, including cancer patients.^33^ The presence of stress, anxiety, and depression can have an impact on overall well‐being and potentially influence cancer treatment adherence and outcomes.

Addressing these impacts requires collaborative efforts from healthcare systems, policymakers, and communities to mitigate disparities and ensure equitable access to cancer care. Strategies may include promoting telemedicine and remote consultations, targeted outreach and education campaigns, improving healthcare infrastructure, addressing social determinants of health, and implementing measures to facilitate safe and timely cancer screenings and treatments during and beyond the pandemic.

## AUTHOR CONTRIBUTIONS

**Benmei Liu:** Conceptualization (equal); formal analysis (lead); methodology (equal); writing – original draft (lead). **Mandi Yu:** Methodology (equal); writing – review and editing (equal). **Jeffrey Byrne:** Data curation (lead); formal analysis (equal). **Katheen A. Cronin:** Methodology (equal); writing – review and editing (equal). **Eric J. Feuer:** Conceptualization (equal); methodology (equal); supervision (equal); writing – review and editing (equal).

## FUNDING INFORMATION

None.

## CONFLICT OF INTEREST STATEMENT

None.

## Supporting information


Table S1.

## Data Availability

The data that support the findings of this study are openly available at the U.S. National Cancer Institute's SEER*Stat Research Plus Limited‐Field Specialized Database at [https://seer.cancer.gov/seerstat/].^25^

## References

[1] World Health Organization . Coronavirus disease (COVID‐19) pandemic. 2024. Accessed February 26, 2024. https://www.who.int/europe/emergencies/situations/covid‐19

[2] Julia Kravchenko MH , Akushevich I . Chronic conditions during the COVID pandemic: mortality patterns in the United States. Innov Aging. 2022;6(Suppl 1):257. doi:10.1093/geroni/igac059.1021

[3] Fedewa SA , Star J , Bandi P , et al. Changes in cancer screening in the US during the COVID‐19 pandemic. JAMA Netw Open. 2022;5(6):e2215490. doi:10.1001/jamanetworkopen.2022.15490 35657622 PMC9166223

[4] Sabatino SA , Thompson TD , White MC , et al. Up‐to‐date breast, cervical, and colorectal cancer screening test use in the United States, 2021. Prev Chronic Dis. 2023;20:E94. doi:10.5888/pcd20.230071 37884318 PMC10625435

[5] Oakes AH , Boyce K , Patton C , Jain S . Rates of routine cancer screening and diagnosis before vs after the COVID‐19 pandemic. JAMA Oncologia. 2023;9(1):145‐146. doi:10.1001/jamaoncol.2022.5481 PMC9673020 36394865

[6] Semprini J , Ranganathan R . The 2020 US cancer screening deficit and the timing of adults' most recent screen: a population‐based cross‐sectional study. Fam Med Community Health. 2023;11(3):e001893. doi:10.1136/fmch-2022-001893 37730268 PMC10510914

[7] Patt D , Gordan L , Diaz M , et al. Impact of COVID‐19 on cancer care: how the pandemic is delaying cancer diagnosis and treatment for American seniors. JCO Clin Cancer Inform. 2020;4:1059‐1071. doi:10.1200/CCI.20.00134 33253013 PMC7713534

[8] Epic Research . Delayed cancer screenings. Epic Research. 2020.Accessed July 18, 2024. https://epicresearch.org/articles/delays‐in‐preventive‐cancer‐screenings‐during‐covid‐19‐pandemic

[9] Mast C , del Rio AM . Delayed cancer screenings—a second look. Epic Research 2020. Accessed July 18, 2024. https://epicresearch.org/articles/delayed‐cancer‐screenings‐a‐second‐look

[10] Centers for Disease Control and Prevention . Behavioral Risk Factor Surveillance System. 2023. Accessed December 5, 2023. https://www.cdc.gov/brfss/index.html

[11] Englum BR , Prasad NK , Lake RE , et al. Impact of the COVID‐19 pandemic on diagnosis of new cancers: a national multicenter study of the veterans affairs healthcare system. Cancer. 2022;128(5):1048‐1056. doi:10.1002/cncr.34011 34866184 PMC8837676

[12] Howlader N , Bhattacharya M , Scoppa S , et al. Cancer and COVID‐19: U.S. Cancer incidence rates during the first year of the pandemic. J Natl Cancer Inst. 2023;116:208‐215. doi:10.1093/jnci/djad205 PMC10852612 37796818

[13] Burus T , Lei F , Huang B , et al. Undiagnosed cancer cases in the US during the first 10 months of the COVID‐19 pandemic. JAMA Oncol. 2024;10(4):500‐507. doi:10.1001/jamaoncol.2023.6969 38386344 PMC10884945

[14] Andraska EA , Alabi O , Dorsey C , et al. Health care disparities during the COVID‐19 pandemic. Semin Vasc Surg. 2021;34(3):82‐88. doi:10.1053/j.semvascsurg.2021.08.002 34642040 PMC8349792

[15] Andrew L , Schmidt ZB , Bhalla S , et al. Cancer care disparities during the COVID‐19 pandemic: COVID‐19 and cancer outcomes study. Cancer Cell. 2020;38(6):769‐770. doi:10.1016/j.ccell.2020.10.023 33176161 PMC7609043

[16] Patel MI , Ferguson JM , Castro E , et al. Racial and ethnic disparities in cancer care during the COVID‐19 pandemic. JAMA Netw Open. 2022;5(7):e2222009. doi:10.1001/jamanetworkopen.2022.22009 35834248 PMC9284331

[17] Fu J , Reid SA , French B , et al. Racial disparities in COVID‐19 outcomes among black and White patients with cancer. JAMA Netw Open. 2022;5(3):e224304. doi:10.1001/jamanetworkopen.2022.4304 35344045 PMC8961318

[18] Semprini J , Pagedar NA , Boakye EA , Osazuwa‐Peters N . Head and neck cancer incidence in the United States before and during the COVID‐19 pandemic. JAMA Otolaryngol Head Neck Surg. 2024;150(3):193‐200. doi:10.1001/jamaoto.2023.4322 38206603 PMC10784997

[19] National Cancer Institute . Registry Groupings in SEER Data and Statistics. 2024. Accessed January 30, 2024. https://seer.cancer.gov/registries/terms.html

[20] Natioanl Cancer Institute . Surveillance, Epidemiology, and End Results Program: Population Characteristics. 2024. Accessed June 21, 2024. https://seer.cancer.gov/registries/characteristics.html

[21] Yu M , Tatalovich Z , Gibson JT , Cronin KA . Using a composite index of socioeconomic status to investigate health disparities while protecting the confidentiality of cancer registry data. Cancer Causes & Control: CCC. 2014;25(1):81‐92. doi:10.1007/s10552-013-0310-1 24178398

[22] National Cancer Institute SEER Program . Incidence Data with Census Tract Attributes Database. 2023. Accessed December 14, 2023. https://seer.cancer.gov/seerstat/variables/countyattribs/census‐tract‐attribs.html

[23] United States Census Bureau . 2010 Census urban and rural classification and urban area criteria. https://www.census.gov/programs‐surveys/geography/guidance/geo‐areas/urban‐rural/2010‐urban‐rural.html

[24] Moss JL , Pinto CN , Srinivasan S , Cronin KA , Croyle RT . Enduring cancer disparities by persistent poverty, rurality, and race: 1990–1992 to 2014–2018. J Natl Cancer Inst. 2022;114(6):829‐836. doi:10.1093/jnci/djac038 35238347 PMC9194626

[25] National Cancer Institute . SEER*Stat Software. 2023. Accessed February 24, 2023. https://seer.cancer.gov/seerstat/

[26] Tiwari RC , Clegg LX , Zou Z . Efficient interval estimation for age‐adjusted cancer rates. Stat Methods Med Res. 2006;15(6):547‐569. doi:10.1177/0962280206070621 17260923

[27] National Cancer Institute . SEER*Stat Tutorials—Step 3: Calculating age‐adjusted rates. 2024. Accessed July 16, 2024. https://seer.cancer.gov/seerstat/tutorials/aarates/step3.html

[28] Midthune DN , Fay MP , Clegg LX , Feuer EJ . Modeling reporting delays and reporting corrections in cancer registry data. J Am Stat Assoc. 2005;100(469):61‐70.

[29] Schenker N , Gentleman JF . On judging the significance of differences by examining the overlap between confidence intervals. The Am Stat. 2001;55:182‐186. doi:10.1198/000313001317097960

[30] Sharpless NE . COVID‐19 and cancer. Science. 2020;368(6497):1290. doi:10.1126/science.abd3377 32554570

[31] Masterson JM , Luu M , Dallas KB , Daskivich LP , Spiegel B , Daskivich TJ . Disparities in COVID‐19 disease incidence by income and vaccination coverage—81 communities, Los Angeles, California, July 2020–September 2021. MMWR Morb Mortal Wkly Rep. 2023;72:728‐731. 10.15585/mmwr.mm7226a5 37384567 PMC10328486

[32] Moynihan R , Sanders S , Michaleff ZA , et al. Impact of COVID‐19 pandemic on utilisation of healthcare services: a systematic review. BMJ Open. 2021;11:e045343. doi:10.1136/bmjopen-2020-045343 PMC7969768 33727273

[33] Xiong J , Lipsitz O , Nasri F , et al. Impact of COVID‐19 pandemic on mental health in the general population: a systematic review. J Affect Disord. 2020;277:55‐64. doi:10.1016/j.jad.2020.08.001 32799105 PMC7413844

