# Supplementary material for: The COVID‐19 pandemic and associated declines in cancer incidence by race/ethnicity and census‐tract level SES, rurality, and persistent poverty status
Source: Cancer Med. 2024 Sep 13;13(17):e70220. doi: 10.1002/cam4.70220 (PMC11393559; doi:10.1002/cam4.70220)
Supplement: Supplementary file 1 — Table S1. [file CAM4-13-e70220-s001.docx]

**Supplement tables for “Impact of the COVID-19 pandemic on the decline of cancer incidence by race/ethnicity and census-tract level SES, rurality, and persistent poverty status” by Liu B, et al**

Supplement Table S1: Delay adjusted, age-adjusted cancer incidence rate (per 100,000 people) in diagnosis years 2015-2019 and 2020 and percent change (PC) between years overall, by race/ethnicity and by SES groups for selected cancer sites (see pages 2-3).

Supplement Table S2: Delay adjusted, age-adjusted cancer incidence rate (per 100,000 people) in diagnosis year 2015-2019 and 2020 and percent change between years by PP status, Rurality and PP cross-by Rurality for selected cancer sites (see pages 4-5).

Table S1: Delay adjusted, age-adjusted cancer incidence rate (per 100,000 people) in diagnosis years 2015-2019 and 2020 and percent change (PC) between years overall, by race/ethnicity and by SES groups for selected cancer sites.

| Cancer sites | **Year** | **Overall** | **Race/Ethnicity** | | | | | **SES Yost Index** | |
| --- | --- | --- | --- | --- | --- | --- | --- | --- | --- |
|  |  |  | NH White | NH Black | NH AIAN (CHSDA) | NH API | Hispanic | Quintile=1 | Quintile=5 |
| **Group 1: Screening is recommended** | | | | | | | | | |
| Breast (Female) | rate 2015-19 | 126.1 (125.9, 126.3) | 139 (138.6, 139.5) | 132.8 (131.8, 133.9) | 119.1 (113.4, 125.1) | 110.9 (109.9, 112) | 102.1 (101.4, 102.9) | 109.7 (109.1, 110.3) | 147.1 (146.6, 147.6) |
|  | rate 2020 | 115.9 (115.4, 116.5) | 130.8 (129.8, 131.8) | 123.6 (121.4, 125.8) | 106.1 (94.4, 118.9) | 103.9 (101.7, 106.2) | 94.8 (93.3, 96.4) | 101.2 (99.9, 102.5) | 136.9 (135.8, 138) |
|  | PC | -8.1 (-8.5, -7.6) | -5.9 (-6.7, -5.1) | -7.0 (-8.7, -5.1) | -10.9 (-21.5, 0.9) | -6.3 (-8.5, -4.1) | -7.2 (-8.8, -5.5) | -7.8 (-9.0, -6.5) | -7.0 (-7.8, -6.1) |
| Prostate (Male) | rate 2015-19 | 112 (111.8, 112.3) | 115.1 (114.7, 115.5) | 193.9 (192.5, 195.4) | 79.7 (74.4, 85.3) | 63.9 (63.0, 64.8) | 92.8 (92, 93.6) | 107.1 (106.5, 107.7) | 123.9 (123.4, 124.4) |
|  | rate 2020 | 104 (103.5, 104.6) | 111.6 (110.7, 112.5) | 179.9 (176.8, 182.9) | 81.6 (70.0, 94.4) | 58.3 (56.5, 60.2) | 81.6 (79.9, 83.3) | 96 (94.7, 97.3) | 122.4 (121.3, 123.5) |
|  | PC | -7.1 (-7.6, -6.6) | **-3.1 (-3.9, -2.2)** | **-7.3 (-9.0, -5.5)** | 2.4 (-13.4, 20.4) | **-8.7 (-11.9, -5.5)** | **-12.1 (-14.0, -10.1)** | **-10.4 (-11.7, -9.1)** | **-1.2 (-2.2, -0.2)** |
| Lung and Bronchus (Female) | rate 2015-19 | 45.3 (45.1, 45.4) | 54.9 (54.7, 55.2) | 46.3 (45.7, 46.9) | 47.7 (44.2, 51.5) | 29.0 (28.5, 29.5) | 23.3 (23.0, 23.7) | 50.1 (49.7, 50.5) | 41.9 (41.6, 42.1) |
|  | rate 2020 | 37.9 (37.6, 38.1) | 47.6 (47.1, 48.1) | 38.3 (37.1, 39.5) | 47.5 (39.7, 56.3) | 24.8 (23.8, 25.9) | 20 (19.3, 20.8) | 43.2 (42.4, 44.0) | 35.8 (35.3, 36.4) |
|  | PC | -16.4 (-17, -15.7) | **-13.3 (-14.38, -12.3)** | **-17.2 (-20, -14.40)** | -0.4 (-18.1, 20.2) | -14.5 (-18.4, -10.5) | -14.1 (-17.5, -10.7) | -13.8 (-15.5, -12.1) | -14.5 (-15.9, -13.0) |
| Lung and Bronchus (Male) | rate 2015-19 | 56.9 (56.7, 57.1) | 64.7 (64.4, 65.0) | 72.6 (71.7, 73.5) | 55.9 (51.4, 60.7) | 45.1 (44.4, 45.9) | 34 (33.5, 34.5) | 75.1 (74.6, 75.6) | 44.7 (44.4, 45.1) |
|  | rate 2020 | 46.7 (46.4, 47.1) | 55.4 (54.8, 56.0) | 60.2 (58.4, 62.0) | 53.4 (44.0, 64.2) | 37.1 (35.6, 38.5) | 26.9 (25.9, 27.9) | 63.7 (62.7, 64.8) | 38.1 (37.5, 38.7) |
|  | PC | -17.8 (-18.5, -17.2) | **-14.4 (-15.5, -13.3)** | **-17.1 (-19.8, -14.4)** | -4.4 (-22.6, 17.0) | -17.8 (-21.3, -14.3) | **-20.8 (-24, -17.6)** | -15.1 (-16.6, -13.6) | -14.8 (-16.3, -13.3) |
| Colon and Rectum (All) | rate 2015-19 | 36.6 (36.5, 36.7) | 38.4 (38.3, 38.6) | 45.1 (44.7, 45.6) | 52.3 (49.5, 55.2) | 31.3 (30.9, 31.8) | 35.1 (34.7, 35.4) | 42.9 (42.6, 43.1) | 33.6 (33.4, 33.8) |
|  | rate 2020 | 31.8 (31.6, 32.0) | 34.3 (34.0, 34.7) | 38.2 (37.3, 39.1) | 51.8 (45.7, 58.5) | 26.6 (25.8, 27.4) | 30.8 (30.2, 31.5) | 37.8 (37.2, 38.3) | 30.1 (29.7, 30.5) |
|  | PC | -13.0 (-13.6, -12.4) | **-10.6 (-11.7, -9.6)** | **-15.4 (-17.5, -13.1)** | -1.0 (-13.7, 13.2) | -15.1 (-17.9, -12.2) | -12.1 (-14.1, -10.0) | -11.9 (-13.3, -10.5) | -10.4 (-11.6, -9.2) |
| Cervix Uteri (Female) | rate 2015-19 | 7.7 (7.6, 7.8) | 7.2 (7.1, 7.3) | 9.2 (8.9, 9.5) | 10.5 (8.8, 12.4) | 6.5 (6.213, 6.7)* | 10.2 (9.9, 10.4) | 12 (11.8, 12.2) | 5.1 (4.99, 5.2)* |
|  | rate 2020 | 6.8 (6.7, 6.9) | 6.3 (6.1, 6.6) | 8.0 (7.5, 8.6) | 8.1 (4.9, 12.5) | 5.7 (5.2, 6.211)* | 9.2 (8.8, 9.7) | 10.6 (10.2, 11.1) | 4.8 (4.6, 5.02)* |
|  | PC | -11.8 (-13.7, -9.9) | -11.6 (-15.3, -7.7) | -12.7 (-19.3, -5.7) | -22.5 (-54.2, 23.6) | -12.3 (-20.7, -3.1) | -9.4 (-14.2, -4.3) | -11.4 (-15.4, -7.4) | -5.9 (-10.5, -1.0) |
| **Group 2: Early detection frequently occurs** | | | | | | | | | |
| Thyroid (Female) | rate 2015-19 | 21.0 (20.9, 21.1) | 23.1 (22.9, 23.3) | 13.1 (12.8, 13.5) | 22.5 (20, 25.2) | 23.7 (23.2, 24.2) | 21.9 (21.6, 22.2) | 18.3 (18.1, 18.6) | 23.8 (23.6, 24) |
|  | rate 2020 | 16.7 (16.4, 16.9) | 18.3 (17.9, 18.7) | 11.1 (10.4, 11.8) | 18.6 (13.7, 24.5) | 18.4 (17.4, 19.3) | 18.3 (17.7, 19) | 15 (14.5, 15.5) | 18.8 (18.4, 19.3) |
|  | PC | -20.6 (-21.7, -19.5) | -20.7 (-22.6, -18.6) | -15.6 (-21, -9.9) | -17.5 (-40.4, 11.8) | -22.5 (-26.7, -18) | -16.3 (-19.4, -13.1) | -18 (-21, -14.9) | -21.1 (-23.1, -19) |
| Thyroid (Male) | rate 2015-19 | 7.5 (7.5, 7.6) | 9.0 (8.9, 9.1) | 3.9 (3.7, 4.1) | 5.6 (4.4, 7.1) | 8.1 (7.8, 8.4) | 6.1 (5.96, 6.3)* | 5.7 (5.5, 5.8) | 9.6 (9.5, 9.8) |
|  | rate 2020 | 6.5 (6.4, 6.6) | 8.0 (7.7, 8.3) | 3.1 (2.7, 3.5) | 6.1 (3.3, 10.3) | 6.4 (5.8, 7.0) | 5.6 (5.2, 5.98)* | 5.1 (4.8, 5.4) | 8.1 (7.8, 8.4) |
|  | PC | -13.6 (-15.5, -11.7) | -11.5 (-14.7, -8.2) | -20.9 (-31.2, -9.5) | 9.1 (-44.4, 95.7) | -21.6 (-29.2, -13.3) | -9.1 (-15.7, -2.1) | -10.4 (-16.1, -4.4) | -15.9 (-19.1, -12.5) |
| Melanoma of the Skin (All) | rate 2015-19 | 20.7 (20.7, 20.8) | 32.2 (32.0, 32.3) | 0.97 (0.91, 1.05)* | 9.4 (8.2, 10.8) | 1.3 (1.2, 1.4) | 4.5 (4.4, 4.6) | 10.3 (10.2, 10.4) | 31.1 (30.9, 31.3) |
|  | rate 2020 | 17.5 (17.4, 17.7) | 28.2 (27.9, 28.6) | 0.9 (0.8, 1.1) | 7.9 (5.5, 10.9) | 1.1 (0.9, 1.3) | 3.9 (3.7, 4.2) | 9.5 (9.2, 9.7) | 26.1 (25.7, 26.4) |
|  | PC | -15.4 (-16.2, -14.6) | -12.2 (-13.3, -11.0) | -4.4 (-20.2, 13.9) | -16.3 (-43.2, 19.4) | -17.7 (-30.8, -2.7) | -12.6 (-18.2, -6.8) | **-8.2 (-11.2, -5.2)** | **-16.1 (-17.3, -14.9)** |
| **Group 3: Other selected sites** | | | | | | | | | |
| Pancreas (All) | rate 2015-19 | 13.02 (12.96, 13.07) | 13.7 (13.6, 13.8) | 16.3 (16, 16.6) | 13.3 (11.9, 14.8) | 10.1 (9.9, 10.4) | 12.1 (11.9, 12.3) | 13.4 (13.3, 13.6) | 13.6 (13.5, 13.7) |
|  | rate 2020 | 12.6 (12.5, 12.7) | 13.5 (13.3, 13.7) | 15.9 (15.4, 16.5) | 16.5 (13.1, 20.4) | 9.7 (9.2, 10.2) | 11.9 (11.5, 12.3) | 13.1 (12.8, 13.4) | 13.7 (13.4, 13.9) |
|  | PC | -3.3 (-4.3, -2.3) | -1.6 (-3.3, 0.1) | -2.3 (-6.2, 1.8) | 23.9 (-3.9, 58.2) | -4.0 (-9.2, 1.5) | -2.0 (-5.7, 2.0) | -2.3 (-4.9, 0.4) | 0.9 (-1.1, 2.9) |
| Liver and Intrahepatic Bile Duct (All) | rate 2015-19 | 9.5 (9.4, 9.5) | 7.6 (7.6, 7.7) | 11.2 (11, 11.5) | 19.8 (18.2, 21.6) | 12.8 (12.5, 13.1) | 15.5 (15.3, 15.7) | 14.2 (14.1, 14.4) | 7.1 (7.0, 7.2) |
|  | rate 2020 | 8.6 (8.5, 8.7) | 7.3 (7.1, 7.4) | 9.5 (9.1, 10) | 17.8 (14.4, 21.7) | 10.6 (10, 11.1) | 14 (13.6, 14.5) | 13.1 (12.8, 13.5) | 6.4 (6.3, 6.6) |
|  | PC | -9.3 (-10.4, -8.1) | -4.9 (-7.2, -2.7) | -15 (-19.2, -10.6) | -10.2 (-28.5, 11.6) | **-17.6 (-21.9, -13.1)** | **-9.5 (-12.7, -6.3)** | -7.6 (-10, -5.1) | -9.6 (-12.2, -7.0) |
| Urinary Bladder (All) | rate 2015-19 | 18.3 (18.2, 18.3) | 22.9 (22.8, 23) | 11.5 (11.3, 11.8) | 13.5 (12, 15.1) | 8.8 (8.6, 9.0) | 10.5 (10.3, 10.7) | 15.1 (14.9, 15.2) | 20.4 (20.3, 20.6) |
|  | rate 2020 | 16.3 (16.2, 16.4) | 21.1 (20.8, 21.4) | 11.1 (10.6, 11.6) | 11.8 (8.9, 15.2) | 8.0 (7.6, 8.5) | 9.2 (8.8, 9.6) | 13.7 (13.4, 14.1) | 18.7 (18.4, 19.0) |
|  | PC | -10.9 (-11.7, -10) | -7.8 (-9.1, -6.5) | -3.7 (-8.5, 1.3) | -13 (-35.4, 15.2) | -8.8 (-14.3, -2.9) | -12.3 (-16.3, -8.3) | -8.8 (-11.2, -6.4) | -8.3 (-9.9, -6.7) |
| Kidney and Renal Pelvis (All) | rate 2015-19 | 17.1 (17, 17.1) | 18.1 (17.9, 18.2) | 19.4 (19.1, 19.7) | 28.1 (26.1, 30.2) | 9.0 (8.8, 9.2) | 19.0 (18.7, 19.2) | 20.0 (19.8, 20.2) | 15.3 (15.2, 15.4) |
|  | rate 2020 | 15.6 (15.5, 15.8) | 17 (16.7, 17.2) | 17.4 (16.8, 18.1) | 27.7 (23.5, 32.6) | 8.3 (7.9, 8.8) | 17.7 (17.2, 18.1) | 18.7 (18.3, 19.1) | 14.2 (13.9, 14.4) |
|  | PC | -8.2 (-9.1, -7.3) | -6.1 (-7.6, -4.6) | -10.2 (-13.6, -6.7) | -1.2 (-17.8, 18) | -7.4 (-12.9, -1.6) | -6.9 (-9.7, -4.1) | -6.3 (-8.4, -4.1) | -7.4 (-9.2, -5.5) |
| Ovary (Female) | rate 2015-19 | 10.5 (10.5, 10.6) | 11.2 (11, 11.3) | 9.4 (9.1, 9.6) | 12.1 (10.3, 14.1) | 9.8 (9.4, 10.1) | 10.6 (10.4, 10.9) | 10.3 (10.1, 10.5) | 11.3 (11.1, 11.4) |
|  | rate 2020 | 9.2 (9.1, 9.4) | 9.8 (9.6, 10.1) | 8.2 (7.7, 8.8) | 10.4 (6.9, 14.9) | 9.4 (8.8, 10.1) | 9.2 (8.7, 9.6) | 9 (8.6, 9.4) | 9.8 (9.5, 10.2) |
|  | PC | -12.3 (-13.8, -10.7) | -12.0 (-14.6, -9.2) | -12.0 (-18.4, -5.1) | -14.2 (-44.4, 27.7) | -3.5 (-10.9, 4.3) | -13.8 (-18.5, -8.8) | -12.5 (-16.5, -8.4) | -12.7 (-15.5, -9.7) |
| Corpus and Uterus, NOS (Female) | rate 2015-19 | 26.9 (26.8, 27) | 28.2 (28, 28.4) | 29.7 (29.26, 30.2)* | 28.8 (26.1, 31.7) | 23.1 (22.6, 23.5) | 26.3 (26.0, 26.7) | 28.1 (27.8, 28.4) | 26.8 (26.6, 27.0) |
|  | rate 2020 | 24.9 (24.7, 25.2) | 26 (25.6, 26.5) | 28.3 (27.3, 29.29)* | 32.8 (26.6, 40.1) | 22.2 (21.2, 23.2) | 25.6 (24.8, 26.3) | 26.3 (25.7, 26.9) | 25.4 (24.9, 25.8) |
|  | PC | -7.4 (-8.4, -6.4) | -7.8 (-9.4, -6.1) | -4.9 (-8.6, -1.1) | 14.1 (-9.5, 42.7) | -3.8 (-8.5, 1.1) | -2.9 (-6.1, 0.4) | -6.2 (-8.7, -3.7) | -5.3 (-7.2, -3.4) |

Note: PCs are bolded if they are significantly different between the counterpart groups. * Two or three digits after the decimal place were kept if either the point estimates share the same value with one of the confidence bounds or two confidence intervals share the same confidence bound value if only keeping one digit after the decimal place.

Table S2: Delay adjusted, age-adjusted cancer incidence rate (per 100,000 people) in diagnosis year 2015-2019 and 2020 and percent change between years by PP status, Rurality and PP cross-by Rurality for selected cancer sites.

|  | **Year** | **Persistent Poverty Status** | | **Rurality** | | **Persistent poverty by Rurality** | | | |
| --- | --- | --- | --- | --- | --- | --- | --- | --- | --- |
| **Cancer sites** |  | PP | Non-PP | All Urban | Rural | PP /All Urban | PP /Rural | Non-PP /All Urban | Non-PP /Rural |
| **Group 1: Screening is recommended** | | | | | | | | | |
| Breast (Female) | rate 2015-19 | 108 (107.2, 108.7) | 131.4 (131.2, 131.7) | 128 (127.7, 128.3) | 124.7 (124, 125.4) | 107.5 (106.7, 108.4) | 108.9 (106.4, 111.5) | 130.4 (130.1, 130.8) | 125.8 (125.1, 126.6) |
|  | rate 2020 | 98.1 (96.6, 99.7) | 123 (122.5, 123.6) | 117.5 (116.8, 118.1) | 119.3 (117.7, 120.8) | 96.2 (94.4, 98.0) | 102 (96.4, 107.8) | 120 (119.3, 120.7) | 120.5 (118.9, 122.1) |
|  | PC | **-9.1 (-10.7, -7.5)** | **-6.4 (-6.9, -5.9)** | **-8.2 (-8.8, -7.7)** | **-4.4 (-5.7, -3.0)** | **-10.5 (-12.3, -8.7)** | -6.4 (-11.9, -0.5) | **-8.0 (-8.6, -7.4)** | **-4.2 (-5.6, -2.9)** |
| Prostate (Male) | rate 2015-19 | 110.0 (109.2, 110.9) | 115.8 (115.5, 116.0) | 113.3 (113.0, 113.6) | 112.8 (112.2, 113.5) | 111.9 (110.9, 112.9) | 99.9 (97.5, 102.3) | 113.4 (113.1, 113.8) | 113.7 (113.0, 114.3) |
|  | rate 2020 | 97.4 (95.7, 99.2) | 110.2 (109.7, 110.8) | 105.6 (104.9, 106.2) | 107.4 (106.0, 108.8) | 98.4 (96.4, 100.4) | 88.9 (83.9, 94.1) | 106.3 (105.7, 107.0) | 108.6 (107.2, 110.0) |
|  | PC | **-11.4 (-13.1, -9.8)** | **-4.8 (-5.3, -4.3)** | **-6.8 (-7.4, -6.2)** | **-4.8 (-6.1, -3.5)** | -**12.1 (-14.0, -10.1)** | -11.0 (-16.4, -5.3) | **-6.3 (-6.9, -5.6)** | **-4.5 (-5.8, -3.1)** |
| Lung and Bronchus (Female) | rate 2015-19 | 45.1 (44.7, 45.6) | 46.6 (46.4, 46.7) | 43.1 (43.0, 43.3) | 56.8 (56.3, 57.2) | 42.2 (41.7, 42.8) | 63.4 (61.6, 65.2) | 43.2 (43.1, 43.4) | 56.3 (55.9, 56.8) |
|  | rate 2020 | 37.7 (36.8, 38.7) | 39.8 (39.5, 40.1) | 36.3 (36.0, 36.7) | 50.5 (49.6, 51.5) | 35.1 (34.1, 36.2) | 54.3 (50.6, 58.2) | 36.5 (36.1, 36.9) | 50.3 (49.4, 51.3) |
|  | PC | -16.3 (-18.6, -14.1) | -14.6 (-15.3, -13.9) | **-15.7 (-16.6, -14.9)** | **-11.0 (-12.7, -9.2)** | **-16.8 (-19.4, -14.1)** | -14.4 (-20.7, -7.7) | **-15.6 (-16.5, -14.7)** | **-10.7 (-12.5, -8.8)** |
| Lung and Bronchus (Male) | rate 2015-19 | 70.5 (69.8, 71.2) | 57.4 (57.2, 57.6) | 54.1 (53.9, 54.3) | 73.3 (72.7, 73.8) | 66.3 (65.5, 67.0) | 96 (93.6, 98.4) | 52.7 (52.5, 53.0) | 71.8 (71.3, 72.3) |
|  | rate 2020 | 58.2 (56.8, 59.5) | 48.2 (47.8, 48.6) | 44.4 (44.0, 44.9) | 63.4 (62.3, 64.5) | 54.7 (53.2, 56.2) | 82.0 (77.2, 87.0) | 43.3 (42.8, 43.7) | 62.2 (61.2, 63.3) |
|  | PC | -17.5 (-19.5, -15.5) | -16.0 (-16.7, -15.3) | **-17.9 (-18.7, -17.0)** | **-13.5 (-15.0, -11.9)** | **-17.5 (-19.9, -14.99)*** | -14.6 (-20.0, -8.9) | **-17.9 (-18.8, -17.0)** | **-13.3 (-14.46, -11.7)*** |
| Colon and Rectum (All) | rate 2015-19 | 41.9 (41.6, 42.3) | 37.2 (37.1, 37.3) | 36.3 (36.2, 36.5) | 41.6 (41.3, 41.9) | 40.6 (40.2, 41.0) | 48.6 (47.4, 49.8) | 35.9 (35.7, 36) | 41.1 (40.8, 41.4) |
|  | rate 2020 | 36.4 (35.7, 37.1) | 33 (32.8, 33.2) | 31.7 (31.5, 31.9) | 37.9 (37.3, 38.5) | 35.3 (34.5, 36.1) | 42.7 (40.2, 45.3) | 31.3 (31, 31.6) | 37.6 (37.0, 38.3) |
|  | PC | -13.3 (-15.1, -11.5) | -11.3 (-11.9, -10.6) | **-12.8 (-13.5, -12.0)** | **-8.8 (-10.3, -7.2)** | **-13.0 (-15.1, -10.9)** | -12.2 (-17.8, -6.3) | **-12.7 (-13.5, -11.9)** | **-8.5 (-10.1, -6.8)** |
| Cervix Uteri (Female) | rate 2015-19 | 12.1 (11.8, 12.3) | 7.5 (7.4, 7.5) | 7.9 (7.8, 8.0) | 8.5 (8.3, 8.7) | 12.3 (12.0, 12.6) | 10.7 (9.8, 11.6) | 7.4 (7.3, 7.5) | 8.3 (8.1, 8.5) |
|  | rate 2020 | 10.7 (10.2, 11.3) | 6.7 (6.6, 6.9) | 7.1 (6.9, 7.2) | 8.3 (7.8, 8.8) | 10.9 (10.2, 11.5) | 11.0 (9, 13.3) | 6.6 (6.4, 6.8) | 8.1 (7.6, 8.6) |
|  | PC | -11.3 (-16.1, -6.2) | -10.0 (-12.0, -7.8) | **-10.9 (-13.1, -8.5)** | **-2.0 (-7.9, 4.2)** | -11.6 (-17.1, -5.9) | 3.2 (-16.8, 26.8) | -10.6 (-13.2, -8.1) | -2.4 (-8.6, 4.1) |
| **Group 2: Early detection frequently occurs** | | | | | | | | | |
| Thyroid (Female) | rate 2015-19 | 18.5 (18.2, 18.8) | 21.9 (21.8, 22.0) | 21.5 (21.4, 21.7) | 21.1 (20.8, 21.4) | 18.4 (18.1, 18.8) | 22.7 (21.4, 24.1) | 22.0 (21.9, 22.1) | 21.0 (20.6, 21.3) |
|  | rate 2020 | 14.5 (13.9, 15.1) | 17.7 (17.5, 18.0) | 16.9 (16.7, 17.2) | 17.0 (16.4, 17.7) | 13.7 (13.0, 14.4) | 21.0 (18.3, 24.0) | 17.4 (17.1, 17.7) | 16.7 (16.1, 17.4) |
|  | PC | -21.7 (-25.3, -18.0) | -19.1 (-20.2, -17.9) | -21.4 (-22.7, -20.1) | -19.4 (-22.7, -15.9) | **-25.9 (-29.8, -21.7)** | **-7.5 (-20.4, 7.1)** | -20.9 (-22.2, -19.5) | -20.2 (-23.6, -16.7) |
| Thyroid (Male) | rate 2015-19 | 5.5 (5.3, 5.7) | 8.0 (7.9, 8.1) | 7.8 (7.7, 7.8) | 7.3 (7.1, 7.4) | 5.4 (5.2, 5.6) | 6.1 (5.5, 6.7) | 8.1 (8, 8.1) | 7.4 (7.2, 7.5) |
|  | rate 2020 | 5.1 (4.7, 5.5) | 6.9 (6.8, 7.1) | 6.7 (6.5, 6.8) | 6.8 (6.4, 7.2) | 5.1 (4.7, 5.6) | 5.2 (3.9, 6.7) | 6.9 (6.7, 7) | 6.9 (6.5, 7.3) |
|  | PC | -6.8 (-14.2, 1.1) | -13.1 (-15.0, -11.1) | -14.2 (-16.4, -11.8) | -6.8 (-12.5, -0.8) | -5.5 (-14.1, 3.8) | -14.5 (-36.4, 13.2) | -14.8 (-17.2, -12.4) | -6.6 (-12.4, -0.4) |
| Melanoma of the Skin (All) | rate 2015-19 | 8.1 (8.0, 8.3) | 22.8 (22.7, 22.8) | 19 (18.9, 19.1) | 25.9 (25.6, 26.1) | 6.2 (6.1, 6.4) | 18.6 (17.8, 19.4) | 20.5 (20.4, 20.5) | 26.4 (26.1, 26.6) |
|  | rate 2020 | 7.0 (6.7, 7.3) | 19.6 (19.4, 19.8) | 15.8 (15.6, 16.0) | 23.9 (23.4, 24.4) | 5.5 (5.2, 5.9) | 15.7 (14.2, 17.4) | 17 (16.8, 17.2) | 24.5 (23.9, 25.0) |
|  | PC | -13.7 (-17.7, -9.4) | -13.8 (-14.6, -13.1) | **-16.7 (-17.6, -15.7)** | **-7.6 (-9.6, -5.5)** | -11.1 (-16.5, -5.5) | -15.5 (-24.4, -5.9) | **-16.9 (-17.9, -15.9)** | **-7.2 (-9.3, -5.1)** |
| **Group 3: Other selected sites** |  |  |  |  |  |  |  |  |  |
| Pancreas (All) | rate 2015-19 | 13.4 (13.2, 13.6) | 13.4 (13.30, 13.4)* | 13.1 (13.1, 13.2) | 13.6 (13.4, 13.7) | 13.6 (13.3, 13.8) | 12.8 (12.2, 13.4) | 13.1 (13.02, 13.2) | 13.6 (13.5, 13.8) |
|  | rate 2020 | 12.9 (12.5, 13.3) | 13.2 (13.0, 13.29)* | 12.8 (12.7, 13.0) | 13.4 (13.1, 13.8) | 12.9 (12.5, 13.4) | 12.4 (11.1, 13.8) | 12.8 (12.6, 12.95) | 13.5 (13.2, 13.9) |
|  | PC | -4.3 (-7.6, -0.9) | -1.5 (-2.6, -0.4) | -2.5 (-3.8, -1.2) | -1 (-3.8, 1.8) | -4.6 (-8.4, -0.7) | -3.5 (-14.3, 8.5) | -2.3 (-3.6, -0.9) | -0.8 (-3.7, 2.1) |
| Liver and Intrahepatic Bile Duct (All) | rate 2015-19 | 15.5 (15.3, 15.7) | 9.20 (9.15, 9.25)* | 10.23 (10.17, 10.29)* | 8.4 (8.3, 8.6) | 16.7 (16.5, 16.9) | 9.7 (9.2, 10.2) | 9.5 (9.4, 9.6) | 8.4 (8.2, 8.5) |
|  | rate 2020 | 14.0 (13.6, 14.4) | 8.5 (8.4, 8.6) | 9.1 (9.0, 9.2) | 8.3 (8.0, 8.6) | 14.9 (14.4, 15.4) | 8.4 (7.4, 9.6) | 8.5 (8.3, 8.6) | 8.3 (8.0, 8.6) |
|  | PC | -9.4 (-12.4, -6.4) | -7.4 (-8.7, -6.2) | **-11.1 (-12.4, -9.7)** | **-1.8 (-5.2, 1.8)** | **-10.9 (-14.2, -7.5)** | -13.0 (-24.7, 0.3) | **-11.0 (-12.5, -9.5)** | **-0.8 (-4.4, 2.9)** |
| Urinary Bladder (All) | rate 2015-19 | 13.5 (13.3, 13.7) | 19.3 (19.2, 19.4) | 17.5 (17.4, 17.6) | 21.9 (21.7, 22.1) | 13 (12.8, 13.2) | 17.5 (16.8, 18.2) | 18 (17.9, 18.1) | 22.2 (22.0, 22.4) |
|  | rate 2020 | 12.5 (12.1, 12.9) | 17.5 (17.4, 17.7) | 15.6 (15.5, 15.8) | 20.5 (20.1, 20.9) | 11.7 (11.3, 12.2) | 17.9 (16.3, 19.5) | 16.1 (15.9, 16.2) | 20.7 (20.3, 21.2) |
|  | PC | -7.7 (-11.0, -4.4) | -9.1 (-10.0, -8.2) | **-10.7 (-11.8, -9.7)** | **-6.3 (-8.5, -4.2)** | -9.6 (-13.4, -5.6) | **2.3 (-7.4, 12.8)** | **-10.9 (-11.9, -9.8)** | **-6.8 (-9.0, -4.6)** |
| Kidney and Renal Pelvis (All) | rate 2015-19 | 19.0 (18.8, 19.2) | 17.4 (17.3, 17.4) | 16.6 (16.6, 16.7) | 19.6 (19.4, 19.8) | 18.3 (18.1, 18.6) | 21.4 (20.6, 22.3) | 16.4 (16.3, 16.5) | 19.5 (19.3, 19.7) |
|  | rate 2020 | 17.3 (16.9, 17.8) | 16.2 (16.1, 16.4) | 15.2 (15.0, 15.4) | 19.1 (18.6, 19.5) | 16.8 (16.3, 17.4) | 17.7 (16.1, 19.4) | 15.0 (14.8, 15.2) | 19.2 (18.7, 19.6) |
|  | PC | -8.7 (-11.4, -5.9) | -6.6 (-7.5, -5.6) | **-8.6 (-9.7, -7.5)** | **-2.9 (-5.3, -0.5)** | **-8.2 (-11.4, -4.8)** | -17.5 (-25.5, -8.8) | **-8.6 (-9.8, -7.5)** | **-1.8 (-4.2, 0.8)** |
| Ovarian (Female) | rate 2015-19 | 10.4 (10.2, 10.6) | 10.9 (10.8, 11) | 10.9 (10.8, 11) | 10.3 (10.1, 10.5) | 10.6 (10.3, 10.8) | 8.5 (7.8, 9.3) | 10.9 (10.8, 11) | 10.4 (10.2, 10.7) |
|  | rate 2020 | 9.0 (8.5, 9.5) | 9.7 (9.5, 9.8) | 9.6 (9.4, 9.8) | 9.4 (9.0, 9.9) | 9.0 (8.5, 9.6) | 9.1 (7.4, 11.0) | 9.6 (9.4, 9.8) | 9.5 (9.0, 9.9) |
|  | PC | -13.5 (-18.5, -8.3) | -11.0 (-12.6, -9.4) | -12.1 (-13.9, -10.2) | -8.5 (-13.1, -3.7) | -14.4 (-20, -8.5) | 6.4 (-14.7, 31.7) | -11.8 (-13.8, -9.8) | -9.3 (-14.0, -4.4) |
| Corpus and Uterus, NOS (Female) | rate 2015-19 | 28.6 (28.3, 29.0) | 27.6 (27.5, 27.7) | 28.1 (28.0, 28.2) | 26.4 (26.1, 26.7) | 29.1 (28.7, 29.5) | 28.5 (27.2, 29.8) | 28 (27.8, 28.1) | 26.2 (25.9, 26.6) |
|  | rate 2020 | 26.2 (25.4, 27.0) | 26.0 (25.8, 26.3) | 26.1 (25.8, 26.4) | 24.2 (23.5, 24.9) | 26.6 (25.7, 27.5) | 28.7 (25.8, 31.9) | 26 (25.7, 26.3) | 23.9 (23.2, 24.6) |
|  | PC | -8.4 (-11.5, -5.3) | -5.7 (-6.7, -4.7) | -7.1 (-8.3, -6.0) | -8.3 (-11.0, -5.5) | -8.6 (-12.1, -5.1) | 0.9 (-10.2, 13.1) | -6.9 (-8.1, -5.7) | -8.9 (-11.7, -6.0) |

Note: PCs are bolded if they are significantly different between the counterpart groups. * Two or three digits after the decimal place were kept if either the point estimates share the same value with one of the confidence bounds or two confidence intervals share the same confidence bound value if only keeping one digit after the decimal place.
